# Cytoprotective IgG antibodies in sera from a subset of patients with AQP4-IgG seropositive neuromyelitis optica spectrum disorder

**DOI:** 10.1038/s41598-021-01294-3

**Published:** 2021-11-09

**Authors:** Lukmanee Tradtrantip, Michael R. Yeaman, A. S. Verkman

**Affiliations:** 1 Departments of Medicine and Physiology, University of California, 1246 Health Sciences East Tower, 513 Parnassus Ave, San Francisco, CA 94143-0521 USA; 2 Department of Medicine, David Geffen School of Medicine, University of California, Los Angeles, CA 90095 USA; 3 Division of Molecular Medicine, Harbor-UCLA Medical Center, Torrance, CA 90502 USA; 4 Lundquist Institute for Biomedical Innovation at Harbor-UCLA Medical Center, Torrance, CA 90502 USA

**Keywords:** Neuroimmunology, Medical research, Neurology

## Abstract

Neuromyelitis optica spectrum disorder (NMOSD) is an autoimmune inflammatory disease of the central nervous system. Most NMOSD patients are seropositive for immunoglobulin G (IgG) autoantibodies against astrocyte water channel aquaporin-4 (AQP4), called AQP4-IgG. AQP4-IgG binding to aquaporin-4 causes complement-dependent cytotoxicity (CDC), leading to inflammation and demyelination. Here, CDC was measured in AQP4-expressing cells exposed to human complement and heat-inactivated sera from 108 AQP4-IgG seropositive NMOSD subjects and 25 non-NMOSD controls. AQP4-IgG positive sera produced a wide range of CDC, with 50% maximum cytotoxicity produced by as low as 0.2% serum concentration. Unexpectedly, 58 samples produced no cytotoxicity, and of those, four sera were cytoprotective against cytotoxic AQP4-IgG. Cytoprotection was found against different cytotoxic monoclonal AQP4-IgGs and NMOSD patient sera, and in primary astrocyte cultures. Mechanistic studies revealed that the protective factor is an IgG antibody that did not inhibit complement directly, but interfered with binding of cytotoxic AQP4-IgG to AQP4 and consequent C1q binding and complement activation. Further studies suggested that non-pathogenic AQP4-IgG, perhaps with altered glycosylation, may contribute to reduced or ineffectual binding of cytotoxic AQP4-IgG, as well as reduced cell-surface AQP4. The presence of natural cytoprotective antibodies in AQP4-IgG seropositive sera reveals an added level of complexity in NMOSD disease pathogenesis, and suggests the potential therapeutic utility of ‘convalescent’ serum or engineered protective antibody to interfere with pathogenic antibody in AQP4-IgG seropositive NMOSD.

## Introduction

Neuromyelitis optica spectrum disorder (NMOSD) is an autoimmune inflammatory disease of the central nervous system that can produce demyelination in optic nerve, spinal cord and brain, and consequent neurological deficit^1–4^. More than 70% of NMOSD patients are seropositive for circulating immunoglobulin G (IgG) autoantibodies directed against extracellular epitopes of astrocyte water channel aquaporin-4 (AQP4), called AQP4-IgG^5,6^. There is strong evidence that AQP4-IgG is pathogenic in seropositive NMOSD by a mechanism that involves AQP4-IgG binding to AQP4 and complement activation, which leads to complement-dependent cellular injury and downstream inflammation, blood–brain barrier disruption, myelin loss and neuronal injury^7–9^. T cells may be involved as well in disease pathogenesis.

AQP4-IgG autoantibodies consist a polyclonal and evolving mixture of anti-AQP4 antibodies that recognize various three-dimensional epitopes on cell surface-exposed, extracellular domains of AQP4^10–12^. AQP4-IgG is mainly of the IgG1 immunoglobulin subclass, with its Fc domain possessing effector functions including complement-dependent cytotoxicity (CDC). CDC is initiated by binding of complement protein C1q to AQP4-IgG, which requires supramolecular clustering of AQP4 tetramers at the plasma membrane^13,14^ as well as clustering of AQP4-bound AQP4-IgG^15^. Eculizumab, a monoclonal antibody inhibitor of complement protein C5, was recently approved for use in reducing clinical relapses in AQP4-IgG seropositive NMOSD^16,17^, supporting a central role of complement activation and CDC in human NMOSD. Additional evidence for a major role of complement in NMOSD pathogenesis includes deposition of activated complement in affected human tissues^7,18,19^ and data in experimental animal models showing NMOSD pathology following exposure to AQP4-IgG and complement^20,21^ which is increased in rodents deficient in complement regulator protein CD59^22,23^. Consistent with these findings, an engineered, high-affinity, anti-AQP4 antibody lacking effector function, called aquaporumab, blocks the binding of pathogenic AQP4-IgG to AQP4, and prevents complement activation and consequent cellular injury and pathological changes^24,25^.

The original purpose of this study was to discover potential correlations between serum cytotoxicity and clinical data in seropositive NMOSD patients, with the goal of evaluating the potential utility of serum cytotoxicity as a biomarker of NMOSD disease progression and drug response. In carrying out studies on sera from 108 unique seropositive NMOSD patients, we discovered, unexpectedly, that a substantial percentage of sera did not produce CDC in AQP4-expressing cells, and of those sera, a subset was cytoprotective when added together with pathogenic AQP4-IgG. The study herein is focused on the discovery and characterization of cytoprotective NMOSD sera.

## Results

### AQP4-IgG seropositive sera induce highly variable CDC in AQP4-expressing cells

**Figure 1:**
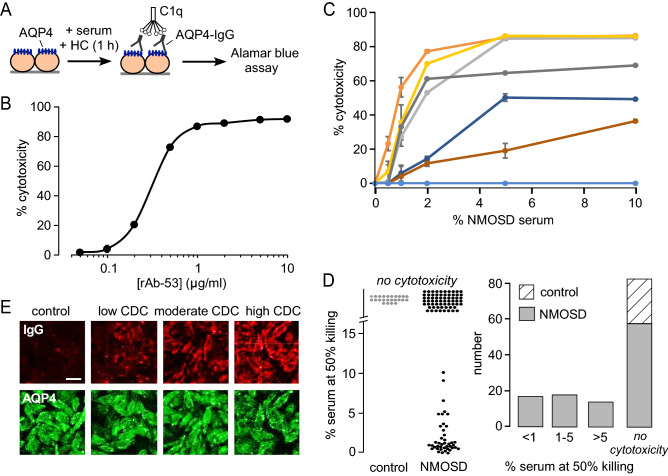
Heterogeneity in complement-dependent cytotoxicity (CDC) produced by sera from AQP4-IgG seropositive NMOSD patients. (**A**) CDC assay, in which AQP4-expressing cells were incubated with human complement (HC) and AQP4-IgG (monoclonal antibody or heat-inactivated patient sera), with Alamar blue readout of cytotoxicity. (**B**) CDC in AQP4-expressing CHO cells produced by different concentrations of monoclonal AQP4-IgG rAb-53 (mean ± S.D., n = 4). (**C**) CDC produced by different percentages of NMOSD patient sera (mean ± S.D., n = 4). Curves for 7 different NMOSD patient sera shown. (**D**) (left) Scatter plot of CDC, expressed as the percentage of serum giving 50% killing, for 108 AQP4-IgG seropositive NMOSD sera and 25 (non-NMOSD) control sera. (Right) Histogram of CDC deduced from data on the left. (**E**) AQP4-IgG seropositivity study in which AQP4-IgG-expressing CHO cells were incubated with test sera, washed, and then immunostained for human IgG (red) and AQP4 (green). Micrographs shown for one control serum and three NMOSD sera which (in experiments as in **C**) produced different levels (low, moderate, high) of CDC.

CDC was assayed in AQP4-expressing cells using an Alamar blue readout in which cells were incubated for 60 min with AQP4-IgG and human complement (Fig. 1A). The AQP4-IgG was in the form of a monoclonal antibody derived from seropositive NMOSD patients, as described^26,27^, or as heat-inactivated NMOSD patient serum. Figure 1B shows CDC produced by the well-characterized NMOSD monoclonal antibody rAb-53 in which increasing rAb-53 concentration produced greater cytotoxicity. The data fitted closely to a single-component model with EC_50_ ~ 0.25 µg/ml rAb-53. In control studies, as reported before^26^, cytotoxicity was not seen with non-NMOSD monoclonal antibodies or with rAb-53 in cells that do not express AQP4 (data not shown).

Similar CDC assays were done with heat-inactivated sera from 108 unique AQP4-IgG seropositive NMOSD patents and 25 unique non-NMOSD controls. Representative cytotoxicity curves are shown in Fig. 1C. A remarkably wide range of cytotoxicity was seen, with some sera producing near maximal cytotoxicity at concentrations of less than 2%. Other sera produced much less cytotoxicity, with some samples producing no cytotoxicity even at 10% concentration. No control serum produced significant cytotoxicity at 10% concentration (not shown). Interestingly, in contrast to the monophasic cytotoxicity curve for rAb-53 in Fig. 1B, the cytotoxicity curves for NMOSD patient sera were generally multi-component and complex, which probably reflects the heterogeneity in complement activation produced by the polyclonal AQP4-IgG in patient sera. For some sera a ‘lag-phase’ was seen at low serum concentrations (yellow and brown curves in Fig. 1C), and some sera showed a biphasic response with reduced cytotoxicity at high concentrations (dark blue curve), raising the possibility that some component(s) of NMOSD patient sera may be cytoprotective. Figure 1D summarizes the cytotoxicity data from all sera, which is shown as a scatter plot and number histogram of sera producing different levels of cytotoxicity quantified as the percentage of serum giving 50% maximum killing. A wide range of values was observed, with 58 of the 108 AQP4-IgG seropositive NMOSD sera showing no significant cytotoxicity.

Figure 1E shows fluorescence micrographs in which the AQP4-expressing cells were incubated with heat-inactivated sera, washed, and then immunostained for human IgG and AQP4. The micrographs confirm AQP4-IgG seropositivity of various sera from AQP4-IgG seropositive NMOSD patients, along with a negative control. No IgG staining was seen for control sera, with different levels of staining seen for sera from AQP4-IgG patients, with greater IgG staining generally seen for sera that produced greater cytotoxicity.

### A subset of AQP4-IgG seropositive sera conferred cytoprotection in the CDC assay

**Figure 2:**
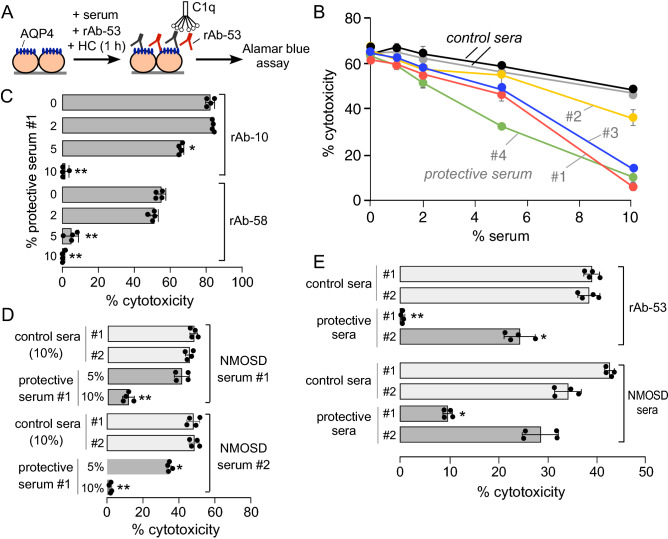
Cytoprotection in a CDC assay conferred by a subset of AQP4-IgG seropositive NMOSD patient sera. (**A**) Cytoprotection assay in which CDC was measured by Alamar blue assay after incubation of AQP4-expressing cells with human complement (HC) and a mixture of cytotoxic AQP4-IgG (such as rAb-53) and heat-inactivated test serum. (**B**) CDC in AQP4-expressing CHO cells as a function of percentage of test NMOSD serum. CDC was produced by 0.5 μg/ml rAb-53 as cytotoxic AQP4-IgG (mean ± S.E.M., n = 4). Data shown for two control sera and four NMOSD patient sera that produced significant cytoprotection. (**C**) CDC measured as in B, but with cytotoxicity produced by monoclonal AQP4-IgGs rAb-10 or rAb-58 (each 5 μg/ml) (mean ± S.D., n = 4, * *P* < 0.05 vs. 0% protective serum by nonparametric Mann–Whitney test). (**D**) CDC measured as in B, with cytotoxicity produced by two NMOSD patient sera (mean ± S.D., n = 4, * *P* < 0.05, ** *P* < 0.01 vs. control sera by Kruskal–Wallis test with Dunn’s multiple comparison). **E.** CDC measured as in B, but with primary cultures of murine astrocytes in which cytotoxicity was produced by rAb-53 or NMOSD patient sera (mean ± S.D., n = 4, * *P* < 0.05 vs. control sera by Kruskal–Wallis test with Dunn’s multiple comparison).

Motivated by the shape of CDC curves produced by NMOSD patient sera, as mentioned above, and the substantial percentage of NMOSD sera that did not produce CDC, we tested the hypothesis that some non-cytotoxic NMOSD sera may contain cytoprotective factor(s). CDC assays were done in which AQP4-expressing cells were incubated with a mixture of cytotoxic AQP4-IgG and non-cytotoxic NMOSD sera (Fig. 2A). Remarkably, 4 out of 58 non-cytotoxic NMOSD sera tested significantly reduced CDC produced by rAb-53, with > 80% reduction in CDC conferred by some sera at 10% concentration (Fig. 2B). On the remaining 54 non-cytotoxic NMOSD sera, small reductions in CDC (range 20–29%) were seen at 10% serum concentration. Small reductions in CDC were also seen with each of the 25 control sera (range 18–26%), with two examples shown in Fig. 2B.

We next investigated whether the cytoprotective sera identified in Fig. 2A were also cytoprotective when studied using different cytotoxic AQP4-IgGs and AQP4-expressing cells. Figure 2C shows cytoprotection against NMOSD monoclonal antibodies rAb-10 and rAb-58, whose AQP4 binding characteristics and epitope specificities differ from those of rAb-53^26^. Figure 2D shows cytoprotection against cytotoxic sera from two NMOSD patients. The cytoprotective action of NMOSD patient sera against various monoclonal and polyclonal AQP4-IgGs indicates that the serum cytoprotective factor is unlikely to be an anti-idiotype antibody. Finally, cytoprotection was also seen using primary murine astrocyte cultures (Fig. 2E) in which AQP4 supramolecular assembly and cellular distribution mimic that in the central nervous system in vivo.

### Clinical correlates of NMOSD patients with cytoprotective sera

Table 1 lists the clinical characteristics of the four AQP4-IgG seropositive NMOSD patients with cytoprotective sera (all female), comparing with averaged data for 54 NMOSD patients with non-cytotoxic, non-cytoprotective sera (~ 90% female), and 50 NMOSD patients with cytotoxic sera. Collectively, no distinguishing characteristics were associated with cytoprotective sera versus the other groups in time from blood draw to last relapse, annualized relapse rate, or time from NMOSD diagnosis to serum collection. Although the numbers are too small for meaningful statistics, interestingly none of the four patients with cytoprotective sera had transverse myelitis symptoms, whereas half of patients in the others groups did. Cerebral and brainstem symptoms appeared to be more frequent in the patients with cytoprotective sera. Drug therapy differed for each of the patients with cytoprotective sera. Because treatment with IVIG at the time of blood draw in subject #4 may have contributed to the measured in vitro cytoprotection, as suggested by in vitro CDC assays with added IVIG^28^, this serum was not studied further.

**Table 1:** Clinical information on NMOSD subjects with cytoprotective sera.

Cytoprotective NMOSD serum subject	Age at blood draw	Time since last relapse (months)	Annualized relapse rate	Time (year) from diagnosis to serum collection	NMOSD symptom(s) history	Drug therapy
#1	31	36.1	0.74	4.5	C, O	MM
#2	33	10.0	0.90	3.5	ON, O	Rituximab
#3	29	63.1	0.33	6.0	B, O	Corticosteroids
#4	52	0^+^	0.87	14.7	O	IVIG
Non-cytotoxic, non-cytoprotective NMOSD sera (N = 54)		38 ± 49	0.73 ± 0.78	7.8 ± 7.6	ON (19), TM (27), B (14), C (8), O (37)	
Cytotoxic NMOSD sera (N = 50)		31 ± 42	1.06 ± 1.15	7.1 ± 7.2	ON (24), TM (25), B (17), C (7), O (34)	

Mean ± S.D., ^+^Blood draw during active relapse.

*B* brainstem symptoms, *C* cerebral symptom, *O* other symptom, *ON* optic neuritis, *TM* transverse myelitis, *MM* mycophenolate mofetil, *IVIG* intravenous immunoglobulin.

### Cytoprotection is conferred by IgG antibody

**Figure 3:**
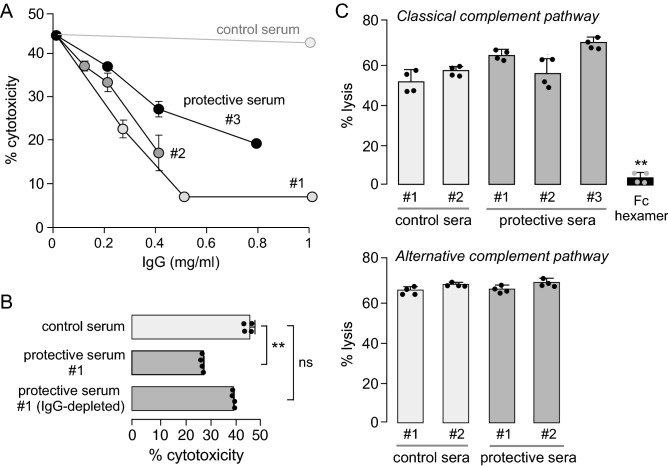
Cytoprotection is conferred by IgG antibodies and does not involve direct complement inhibition. (**A**) CDC measured in AQP4-expressing CHO cells, as in Fig. 2A, but using purified IgG isolated from NMOSD or control sera (mean ± S.D., n = 4). (**B**) CDC measured as in Fig. 2A, but using IgG-depleted cytoprotective NMOSD sera (mean ± S.D., n = 4, ** P < 0.01 vs. control serum, ns not significant, Kruskal–Wallis test with Dunn’s multiple comparison). (**C**) Activity of the classical (top) and alternative (bottom) complement pathways measured in erythrocyte lysis assays (mean ± S.D., n = 4, Kruskal–Wallis test with Dunn’s multiple comparison). Control or cytoprotective sera was present at 10% concentration. Differences not significant. Fc hexamer used as an inhibitor of the classical complement pathway.

In concept, cytoprotection might be conferred by various factors in NMOSD patient sera including antibodies of different classes, non-antibody proteins, or potentially non-protein components such as lipids, small molecules, or electrolytes. To investigate whether the cytoprotective component is an IgG antibody, CDC assays were done using IgG purified from cytoprotective and control sera. Figure 3A shows efficient cytoprotection by IgGs purified from three cytoprotective sera, with 50% cytoprotection produced by 0.2–0.4 mg/ml IgG. Cytoprotection was not seen with IgG identically purified from control serum. Cytoprotection was greatly reduced using IgG-depleted cytoprotective NMOSD serum (Fig. 3B), supporting the conclusion that the cytoprotective factor is an IgG-class antibody.

### Cytoprotective sera do not inhibit complement directly

Possible direct inhibition of the classical or alternative complement activation pathways by cytoprotective sera was tested using standard erythrocyte hemolysis assays in which complement was activated by IgM in antibody-sensitized sheep erythrocytes (classical pathway) or spontaneously activated in rabbit erythrocytes (alternative pathway). Cytoprotective sera, at a concentration conferring strong cytoprotection in the AQP4-IgG CDC assay, did not produce significant inhibition of erythrocyte hemolysis by the classical or alternative complement pathways (Fig. 3C). The cytoprotective mechanism therefore does not involve direct complement inhibition.

### Cytoprotective sera reduce binding of pathogenic AQP4-IgG and C1q to AQP4-expressing cells

Complement activation involves binding of pathogenic AQP4-IgG to AQP4 at the cell surface followed by binding of C1q to the Fc region of AQP4-IgG. Factors in protective sera could interfere with binding of pathogenic AQP4-IgG by, for example, a competitive mechanism^24^, or interfere with C1q binding to AQP4-IgG, by, for example, interfering with supramolecular clustering of AQP4 or AQP4-IgG^13,15,29^. The next experiments address whether cytoprotective IgGs bind to AQP4 and whether binding of pathogenic AQP4-IgG and C1q are reduced in AQP4-expressing cells following incubation with cytoprotective sera.

**Figure 4:**
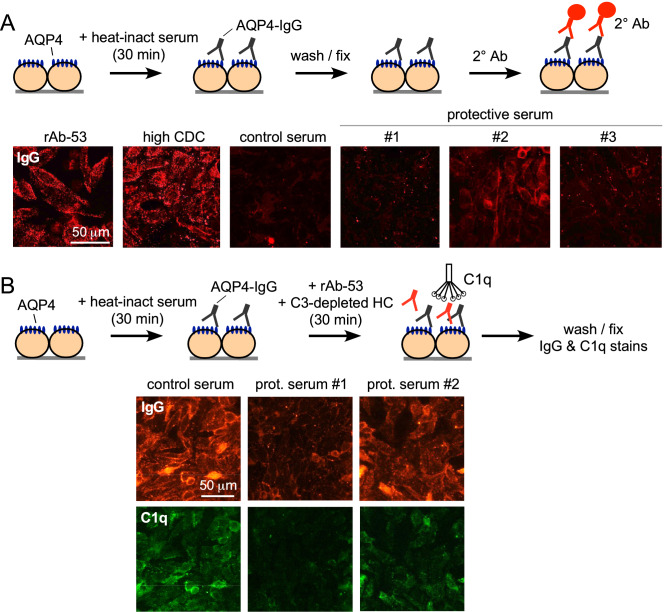
Cytoprotective sera reduces binding of pathogenic AQP4-IgG and C1q at the cell surface. (**A**) Cytoprotective serum binding study. AQP4-expressing CHO cells were incubated with 10% heat-inactivated cytoprotective or control sera (or 5 µg/ml rAb-53), followed by fluorescent anti-human IgG secondary antibody. (**B**) C1q and pathogenic AQP4-IgG (rAb-53) binding. AQP4-expressing CHO cells were incubated with heat-inactivated cytoprotective or control sera followed by 1 µg/ml rAb-53 and 10% C3-deficient human complement, and then stained for human IgG (red) and C1q (green).

Binding of cytoprotective IgGs to AQP4-expressing cells was measured after incubation of cells with serum, washing, and addition of fluorescent anti-human IgG secondary antibody (Fig. 4A). As positive controls, strong cell surface binding (red fluorescence) was seen with rAb-53 and cytotoxic NMOSD sera (labeled ‘high CDC’). Staining was not seen with five control (non-NMOSD) sera, one of which is shown in the figure (‘control serum’). The three cytoprotective sera showed minimal to moderate staining of AQP4-expressing cells (Fig. 4A, right panels), which was absent in non-transfected cells (not shown). These experiments further support the presence of AQP4-binding IgG(s) in cytoprotective NMOSD sera. However, a caveat in this experiment is that AQP4-IgGs with weak binding and rapid off rates would not be detected because of the requisite washing step, so that the observed staining may substantially underestimate actual binding.

Binding of pathogenic AQP4-IgG and C1q was measured in AQP4-expressing cells incubated with heat-inactivated cytoprotective or control sera for 30 min, followed by addition of rAb-53 and C3-depleted complement, the latter providing a source of C1q but without producing cytotoxicity (Fig. 4B). A red fluorescent anti-human IgG secondary antibody revealed bound pathogenic AQP4-IgG (rAb-53). Compared to control serum, reduced rAb-53 binding was seen in the presence of protective NMOSD sera. Though the fluorescent secondary antibody also detects bound IgGs in the cytoprotective sera, little interference with the assay is expected with cytoprotective sera because of their apparent minimal binding (as seen in panel A). C1q binding (green fluorescence) was also substantially reduced with cytoprotective sera compared with the control serum, consistent with reduced rAb-53 binding. The reduced C1q binding is consistent with reduced CDC, as observed.

**Figure 5:**
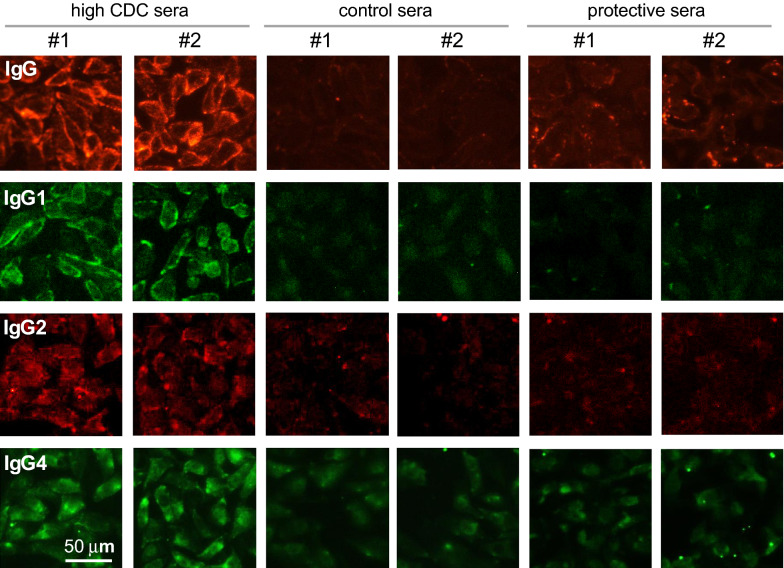
Absence of AQP4-bound IgG2 and IgG4 subclass antibodies in cytoprotective sera. AQP4-expressing CHO cells incubated for 60 min with 10% control (no cytotoxicity) sera, cytoprotective sera, or high cytotoxicity sera. Bound antibody was revealed using fluorescent secondary antibodies selective for total IgG, IgG1, IgG2 and IgG4. Immunofluorescence micrographs representative of 2 sets of studies.

The possibility that cytoprotective sera may contain AQP4-IgG antibodies of the IgG2 or IgG4 subclasses, which lack CDC effector function and hence their presence could confer cytoprotection, was investigated using fluorescent, IgG subclass-selective secondary antibodies. AQP4-expressing cells were incubated with 10% test sera for 60 min, following by washing and immunostaining (Fig. 5). As a positive control, strong total IgG and IgG1 immunofluorescence was seen with two NMOSD sera that produced high cytotoxicity (labeled ‘high CDC sera’). Positive immunofluorescence was also seen for IgG2 and IgG4, albeit weaker, consistent with the presence of multiple AQP4-IgG subclasses as reported previously^30^. Weak total IgG immunofluorescence was seen for the cytoprotective sera comparing to the control sera, though no immunofluorescence was seen for each of the IgG subclasses. These results suggest little AQP4-bound IgG2  or IgG4, subject to the caveat that low affinity antibodies may be removed by the wash step.

### Cytoprotective sera reduces apparent cell-surface AQP4 expression

**Figure 6:**
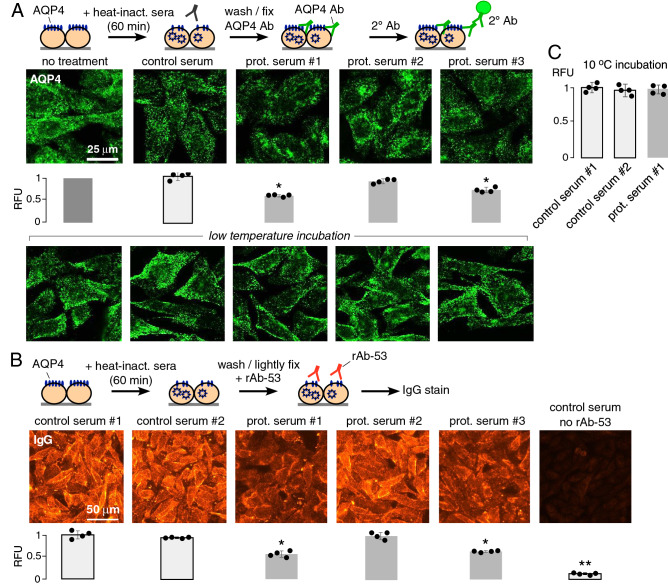
Cytoprotective sera reduces apparent cell-surface AQP4 expression without affecting AQP4 clustering. (**A**) AQP4 immunofluorescence in AQP4-expressing CHO cells after 60 min incubation with 10% heat-inactivated control or cytoprotective NMOSD sera (or no treatment), followed by washing, fixation, cell permeabilization, and staining with C-terminal anti-AQP4 antibody and fluorescent secondary antibody (top). Total cellular fluorescence summarized below each fluorescence micrograph (mean ± S.D., n = 4, * *P* < 0.05, ** *P* < 0.01 vs. control serum by nonparametric Mann–Whitney test). Identical experiments except with the 60 min incubation done at 0 °C to block endocytosis and intracellular trafficking (bottom micrographs). (**B**) Cell-surface AQP4 assay. AQP4-expressing cells were incubated with 10% control or cytoprotective sera, then washed, lightly fixed, and then stained with 0.5 μg/ml rAb-53 and fluorescent anti-human IgG secondary antibody to reveal cell-surface AQP4. Total cellular fluorescence summarized on the bottom (mean ± S.D., n = 4, * *P* < 0.05, ** *P* < 0.01 vs control sera by Kruskal–Wallis test with Dunn’s multiple comparison). (**C**) Experiment as in B. but initial 60 min incubation with sera done at 10 °C to inhibit endocytosis (mean ± S.D., n = 4, differences not significant by Kruskal–Wallis test with Dunn’s multiple comparison).

The reduced binding of pathogenic AQP4-IgG and C1q at the cell surface with cytoprotective sera could be a consequence of reduced cell-surface AQP4, which might be caused by cellular AQP4 internalization. High-magnification confocal micrographs in Fig. 6A (top) show AQP4 immunofluorescence after 60 min exposure to control or cytoprotective sera (or no treatment) as measured using a C-terminal anti-AQP4 antibody in fixed, permeabilized cells. Untreated cells show considerable intracellular fluorescence in a vesicular pattern along with cell-surface fluorescence. A similar staining pattern was found with control sera as seen in the example provided. However, an altered staining pattern, with partial AQP4 accumulation in perinuclear structures, which have the characteristic appearance of endolysosomal bodies, was seen with cytoprotective sera #1 and #3. Total cellular fluorescence was significantly reduced with these two cytoprotective sera (bar graphs beneath micrographs), suggesting AQP4 protein degradation. Perinuclear AQP4 accumulation was not seen when the 60 min serum incubation was done at reduced temperature to block AQP4 cellular internalization and intracellular trafficking (Fig. 6A, bottom panels).

To quantify cell surface-exposed AQP4 more directly, cells were incubated for 60 min with cytoprotective or control sera, fixed, and then stained with rAb-53 and fluorescent anti-human IgG secondary antibody (Fig. 6B). Reduced rAb-53 immunofluorescence was seen in cells exposed to cytoprotective sera #1 and #3 compared with the two control sera. No immunofluorescence was seen in cells exposed to control sera in the absence of rAb-53. This experiment provides evidence for reduced cell-surface AQP4 expression following exposure to some cytoprotective sera, consistent with the study in Fig. 5A. Noted caveats in this experiment, however, include the preferential binding of rAb-53 to AQP4 aggregates over AQP4 tetramers^26^ and additive fluorescence from AQP4-IgGs in cytoprotective sera, which may account for the high fluorescence with protective serum #2. Lastly, the study in Fig. 6B was repeated but with the initial 60 min incubation with protective sera done at reduced temperature to block endocytosis. At reduced incubation temperature the protective serum did not significantly reduce cell-surface AQP4 expression (Fig. 6C), supporting the conclusion that serum-induced AQP4 internalization may be in part responsible for cytoprotection by some sera.

## Discussion

We found a broad range of CDC produced by AQP4-IgG seropositive NMOSD patient sera, including many sera that did not produce CDC, and made the unanticipated discovery that a subset of non-pathogenic sera confer cytoprotection in CDC assays in AQP4-expressing cells when added together with cytotoxic AQP4-IgG. The diversity in CDC produced by NMOSD patient sera is likely the consequence of the highly heterogeneous, polyclonal mixture of AQP4-IgGs in NMOSD sera that differ in their AQP4 binding affinity and specificity, epitope recognition, and ability to cluster^10,15,26,31^, and hence in their ability to bind C1q and activate complement. The heterogenous mixture of AQP4-IgGs is expected over time to mature and evolve through affinity maturation, antigen-spreading and perhaps altered glycosylation. Thus, it is biologically plausible that sera could be more cytotoxic or cytoprotective at different times in the NMOSD disease course, which may correlate with periods of relapse and remission.

A consequential implication of our findings is that AQP4-IgG seropositivity alone provides only a limited description of NMOSD disease activity, as evidenced by the generally poor correlations reported between disease activity and AQP4-IgG antibody titer^32,33^. Assays of serum-induced CDC, taken together with seropositivity data, may improve correlations between disease activity and laboratory data. Further, the current findings support intriguing potential clinical applications. One application is the use of a high-affinity and non-cytotoxic AQP4-binding antibody to interfere with pathogenic IgG in NMOSD, which is the basis for development of aquaporumab, an early-stage biologic with protective efficacy in pre-clinical studies^24,25^. Our results also support the potential therapeutic utility of ‘convalescent’ serum from NMOSD patients having cytoprotective antibodies, or perhaps monoclonal antibodies from such patients generated by genetic means as has been done for cytotoxic AQP4-IgGs^27^.

**Figure 7:**
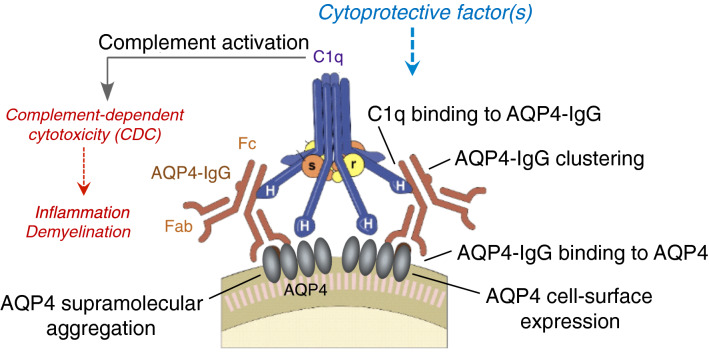
Potential mechanisms of cytoprotective IgG antibodies. Potential sites of action of cytoprotective IgGs include interference with complement activity, AQP4-IgG binding to cell-surface AQP4, supramolecular clustering of AQP4 or AQP4-IgG, and AQP4 cell-surface expression.

Multiple factors act in concert to produce cytotoxicity in AQP4-expressing cells following their exposure to AQP4-IgG and complement, and hence several cytoprotective mechanisms are possible. Experiments using purified IgG and IgG-depleted NMOSD serum indicated that the cytoprotective factor is an IgG-class antibody. CDC in AQP4-expressing cells requires cell-surface exposure of AQP4 supramolecular aggregates, AQP4-IgG binding to the aggregates, AQP4-IgG clustering, C1q binding to the AQP4-IgG clusters, and activation of the classical complement pathway, which ultimately, through the action of a large set of complement proteins and regulatory factors, results in formation of a membrane attack complex and cell injury (Fig. 7)^34,35^. Complement activation also generates anaphylatoxins that recruit and activate leukocytes and lymphocytes to produce a targeted inflammatory milieu. The experiments herein indicate that the cytoprotective factor does not inhibit the classical or alternative complement pathways directly, but interferes with AQP4-IgG binding at the cell surface and consequent C1q binding and complement activation. Reduced AQP4-IgG binding could result from reduced cell-surface exposure of AQP4, by an endocytic internalization mechanism as has been shown for some AQP4-IgGs^36^, by alteration of AQP4 supramolecular aggregation, and/or by prevention of AQP4-IgG binding to AQP4 by a competitive or other mechanism. Experiments herein are consistent with a cytoprotective mechanism involving reduced cell-surface exposure of AQP4 aggregates, and perhaps, for some sera, the presence of a competing, non-pathogenic AQP4-IgG. IgG2 or IgG4 subclass AQP4-binding antibodies were not detected, which lack CDC effector function and hence could be cytoprotective. However, based on precedents in other humoral autoimmune disorders^37,38^, AQP4-IgGs may be present with altered Fc glycosylation and reduced CDC effector function^39^. Other possibilities, though unlikely, include the presence of protective AQP4-IgG-containing immune complexes in cytoprotective sera, or perhaps Fab fragments of AQP4-IgG that interfere with binding of cytotoxic AQP4-IgG to AQP4. Further studies will be required to clarify the cytoprotective mechanisms, which may be challenging because of the complex and heterogeneous composition of serum IgG antibodies, some of which may have relatively low binding affinity and hence rapid dissociation kinetics to AQP4 or potentially neighboring AQP4-associated proteins.

Additional limitations of this study are noted. As the experiments utilized human sera, potential variability in sample handling and storage could affect results. However, this is unlikely to be a source of significant variability because sera were obtained, processed and stored under standardized, uniform conditions. Furthermore, CDC testing of several cytotoxic sera done 6 months apart showed minimal (< 10%) differences in cytotoxicity curves (not shown). Because only single specimens were available from subjects with cytoprotective serum, our data do not address whether serum was cytotoxic in the same subject earlier or later in their disease course, though the patients’ history of prior active NMOSD disease suggests that serum was cytotoxic at some earlier time. Longitudinal studies are warranted addressing potential correlations between cytoprotective vs. cytotoxic antibody relative to disease activity. Finally, we acknowledge that the limited quantities of sera available precluded definitive mechanistic studies which would require larger-scale IgG purifications, such as analysis of IgG glycosylation or isolation of IgG subfractions with different AQP4 binding properties or cytotoxicity.

In conclusion, a subset of AQP4-IgG seropositive NMOSD patients appear to have circulating IgG antibodies in their serum that are cytoprotective against complement-induced injury to AQP4-expressing cells, a primary disease-causing event in AQP4-IgG seropositive NMOSD. Cytoprotective antibodies, which could be studied only using NMOSD patient sera that did not by themselves produce CDC, may be present as well in most or all NMOSD patient sera, and in theory could account for periods of disease activity vs. quiescence. However, isolation and characterization of cytoprotective antibodies in NMOSD patient sera that by themselves produce CDC is likely to be challenging. Identification and generation of monoclonal antibodies from plasma cells of NMOSD subjects with cytoprotective antibody clones may be informative to further clarify cytoprotective mechanisms, which may be multifactorial and differ from patient to patient and over time in the same patient. We speculate that cytoprotective autoantibodies may be present in other antibody-driven autoimmune disorders as well^40^. Lastly, our data support the concept that cytoprotective antibodies may enable novel therapeutic agents or strategies, such as use of convalescent sera or engineered protective antibodies or antibody fragments, for treatment of acute disease exacerbations or prevent relapses in patients with AQP4-IgG seropositive NMOSD.

## Methods

### NMOSD and control sera

Sera from confirmed AQP4-IgG seropositive NMOSD patients and non-NMOSD control sera were provided by the CIRCLES Repository of the Guthy-Jackson Charitable Foundation. Sera were stored at − 70 °C and heat-inactivated at 56 °C for 30 min before use to abolish any intrinsic complement activity.

### Cell culture

Chinese hamster ovary (CHO) cells stably expressing M23-AQP4 (CHO-AQP4 cells) were generated by stable transfection as described^41^. CHO cells were cultured at 37 °C in 5% CO_2_ 95% air in F-12 Ham’s Nutrient Mixture medium supplemented with 10% fetal bovine serum, 100 U/ml penicillin and 100 μg/ml streptomycin. Geneticin (200 μg/ml) was used as selection antibiotic. Primary astrocyte cultures were generated from cerebral cortex of neonatal mice as described^22^.

### CDC assays

CHO-AQP4 cells were plated onto 96-well microplates at 25,000 cells/well and grown for 18–24 h to confluence. Cells were washed with PBS and incubated 60 min with rAb-53 (0.5–10 μg/ml) or heat-inactivated patient serum (1, 2, 5 and 10%) together with 5% human complement (Innovative Research, Novi, MI) in 50 μl live-cell buffer (PBS containing 6 mM glucose, 1 mM sodium pyruvate) at 27 °C. To measure cytotoxicity, cells were washed twice with PBS and incubated with 50 μl of a 20% Alamar blue solution (Invitrogen) for 45 min at 37 °C. Cytotoxicity was measured from resorufin fluorescence (excitation/emission 560/590 nm). For normalization, 0.1% Triton-X treatment gave 100% cytotoxicity and human complement alone gave 0% cytotoxicity.

To test the protective effect of patient sera, CHO-AQP4 cells were incubated for 1 h at 27 ºC with heat-inactivated patient sera, 0.5 μg/ml rAb-53 and 5% human complement. Cytotoxicity were measured using the Alamar blue assay. In some experiments, monoclonal AQP4-IgGs rAb-10 or rAb-58^26^ (each 5 μg/ml) or seropositive NMOSD patient sera (0.5 or 1%), added together with 2% or 10% human complement, were used to induced cytotoxicity. For CDC assays in primary mouse astrocyte cultures, 10% test sera was added together with 20 μg/ml rAb-53 or 10% NMOSD patient serum and 5% human complement and incubated for 1 h. Cells were washed and cytotoxicity was measured by Alamar blue assay.

### Immunofluorescence

To study AQP4-IgG binding in patient sera CHO-AQP4 cells were plated onto 8-well glass chamber slides and grown for 18–24 h to confluence. After blocking with 1% BSA in live-cell buffer, cells were incubated with 10% heated-inactivated patient serum for 1 h at room temperature. Cells were washed with PBS and briefly fixed with 4% paraformaldehyde, then incubated with goat anti-human IgG-conjugated Alexa Fluor-555 secondary antibody (1:500, Invitrogen) for 30 min. Cells were rinsed extensively with PBS and mounted on coverglasses with ProLong Gold antifade mounting reagent (Thermo Fisher Scientific, Rockford, IL). For C1q immunostaining, 10% C3-deficient human complement (Complement Technology Inc., Tyler, TX) and 1 μg/ml rAb-53 were added together to CHO-AQP4 cells after 30 min preincubation with 10% patient sera. C1q was labeled with FITC-conjugated rabbit anti-human C1q antibody (1:50, Abcam, Cambridge, MA) and human IgG was labeled with goat anti-human IgG-conjugated Alexa Fluor-555 secondary antibody (1:500). Immunofluorescence was imaged by a Leica DM 4000B microscope (Wetzlar, Germany). IgG1, IgG2, and IgG4 immunostaining were done in CHO-AQP4 cells after 1 h incubation with 10% patient sera. IgG1 was labeled using mouse anti-human IgG1 conjugated with Alexa Fluor-488 (1:100, Invitrogen). IgG2 and IgG4 were labeled using mouse anti-human IgG2 antibody (1:100, Invitrogen) or rabbit anti-human IgG4 antibody (1:100, Invitrogen), followed by goat anti-rabbit IgG-conjugated Alexa-Fluor-488, and goat anti-mouse IgG-conjugated Alexa-Fluor-555 secondary antibody (1:200, Invitrogen).

To study the effect of patient sera on AQP4 expression, CHO-AQP4 cells were treated with 10% heat-inactivated patient sera for 1 h, and cells were then fixed in 4% PFA for 15 min and permeabilized with 0.1% Triton-X for 10 min. After blocking again with blocking buffer, cells were incubated with a C-terminus rabbit anti-AQP4 antibody (anti-AQP4 249-323 antibody, 1:1,000, Alomone Labs, Jerusalem, Israel) for 1 h, followed by goat anti-rabbit IgG-conjugated Alexa Fluor-488 secondary antibody (1:500, Invitrogen). AQP4 immunofluorescence was visualized on a Nikon confocal microscope using a × 100 oil immersion lens.

To quantify cell-surface AQP4 expression, CHO-AQP4 cells were incubated with 10% heat-inactivated sera for 1 h, then washed and briefly fixed with 4% PFA for 5 min. Cells were then incubated with rAb-53 (0.5 μg/ml) for 30 min, followed by goat anti-human IgG-conjugated Alexa Fluor-555 secondary antibody (1:500). Relative fluorescence was quantified using Image J software (NIH) as the (background-subtracted) fluorescence signal relative to control serum-treated cells.

### IgG isolation and depletion

IgG was purified from patient sera and concentrated using a Melon gel IgG purification kit (Thermo Fisher Scientific, Rockford, IL) and Amicon ultra centrifugal filter units (Millipore, Billerica, MA). IgG concentrations were quantitated by human-IgG ELISA kit (Innovative Research). To deplete IgG in patient sera, Dynabeads Protein G (Invitrogen) were added to patient sera and incubated with rotation for 10 min at room temperature. Beads were removed magnetically and supernatants were used for CDC assays.

### Erythrocyte hemolysis assays of complement activity

Hemolysis assays using IgM-coated sheep red blood cells (for assay of classical complement pathway) and uncoated rabbit red blood cells (for assay of alternative pathway) were performed according to manufacturers’ instructions (Complement Technology Inc., Tyler, TX). Briefly, 2.5 × 10^7^ sheep red blood cells were incubated with specified concentrations of complement for 1 h at 37 °C in a total volume of 0.25 ml. 2.5 × 10^7^ rabbit red blood cells were incubated with complement in Ca^2+^/Mg^2+^ free buffer containing 5 mM MgEGTA (total volume 0.25 mL) for 30 min at 37 °C. Cells were then centrifuged and supernatant absorbance was measured at 541 nm. Control sera was used as negative control and recombinant IgG1 Fc hexamer^42^ was used as positive control.

### Statistical analysis

Data are presented as mean ± S.D. Statistical comparisons were made using the non-parametric Mann–Whitney test when comparing two groups. A *P* value of 0.05 or less was considered significant. For a comparison of more than two groups, the Kruskal–Wallis test with Dunn’s multiple comparison was used (*P* value corrected for multiple comparisons).

## Data Availability

All data supporting the conclusions of this manuscript are provided in the text and figures. Original data will be made available on request.

## Abbreviations

AQP4: Aquaporin-4
AQP4-IgG: Aquaporin-4-immunoglobulin G
CDC: Complement-dependent cytotoxicity
CHO: Chinese hamster ovary
HBSS: Hank’s balanced salt solution
HC: Human complement
IgG: Immunoglobulin G
NMOSD: Neuromyelitis optica spectrum disorder
PFA: Paraformaldehyde
rAb: Recombinant NMOSD monoclonal antibody
